# Influence of genetic variants on toxicity to anti-tubercular agents: a systematic review and meta-analysis (protocol)

**DOI:** 10.1186/s13643-017-0533-4

**Published:** 2017-07-13

**Authors:** Marty Richardson, Jamie Kirkham, Kerry Dwan, Derek Sloan, Geraint Davies, Andrea Jorgensen

**Affiliations:** 1 Department of Biostatistics, University of Liverpool, Whelan Building, Liverpool, L69 3GB UK; 2 Cochrane Editorial Unit, London, SW1Y 4QX UK; 3 School of Medicine, University of St Andrews, St. Andrews, KY16 9TF UK; 4 Department of Clinical Infection, Microbiology and Immunology, University of Liverpool, Liverpool, L69 3GB UK

**Keywords:** Tuberculosis, Pharmacogenetics, Toxicity, Meta-analysis

## Abstract

**Background:**

Tuberculosis patients receiving anti-tuberculosis treatment may experience serious adverse drug reactions, such as hepatotoxicity. Genetic risk factors, such as polymorphisms of the *NAT2*, *CYP2E1* and *GSTM1* genes, may increase the risk of experiencing such toxicity events. Many pharmacogenetic studies have investigated the association between genetic variants and anti-tuberculosis drug-related toxicity events, and several meta-analyses have synthesised data from these studies, although conclusions from these meta-analyses are conflicting. Many meta-analyses also have serious methodological limitations, such as applying restrictive inclusion criteria, or not assessing the quality of included studies. Most also only consider hepatotoxicity outcomes and specific genetic variants. The purpose of this systematic review and meta-analysis is to give a comprehensive evaluation of the evidence base for associations between any genetic variant and anti-tuberculosis drug-related toxicity.

**Methods:**

We will search for studies in MEDLINE, EMBASE, BIOSIS and Web of Science. We will also hand search reference lists from relevant studies and contact experts in the field. We will include cohort studies, case–control studies and randomised controlled trials that recruited patients with tuberculosis who were either already established on anti-tuberculosis treatment or were commencing treatment and who were genotyped to investigate the effect of genetic variants on any anti-tuberculosis drug-related toxicity outcome. One author will screen abstracts to identify potentially relevant studies and will then obtain the full text for each potentially relevant study in order to assess eligibility. At each of these stages, a second author will independently screen/assess 10% of studies. Two authors will independently extract data and assess the quality of studies using a pre-piloted data extraction form. If appropriate, we will pool estimates of effect for each genotype on each outcome using meta-analyses stratified by ethnicity.

**Discussion:**

Our review and meta-analysis will update and add to the existing research in this field. By not restricting the scope of the review to a specific drug, genetic variant, or toxicity outcome, we hope to synthesise data for associations between genetic variants and anti-tuberculosis drug-related toxicity outcomes that have previously not been summarised in systematic reviews, and consequently, add to the knowledge base of the pharmacogenetics of anti-tuberculosis drugs.

**Systematic review registration:**

PROSPERO CRD42017068448

**Electronic supplementary material:**

The online version of this article (doi:10.1186/s13643-017-0533-4) contains supplementary material, which is available to authorized users.

## Background

Tuberculosis (TB) is one of the most important challenges in global health at the present time. This infectious disease ranks alongside human immunodeficiency virus as a leading cause of death worldwide; there were an estimated 1.4 million TB deaths in 2015 [1]. The World Health Organization (WHO) currently recommends a combination of four first-line drugs for individuals with drug-susceptible TB: isoniazid, rifampicin, ethambutol and pyrazinamide [1].

TB patients receiving this regimen may experience serious adverse drug reactions, such as anti-tuberculosis drug-induced hepatotoxicity (ATDH), which causes a strain on health care providers due to high morbidity, mortality and increased treatment costs [2, 3]. Reported incidence rates of ATDH among patients treated with standard multidrug treatment vary widely from 2 to 28%, depending on the regimen given and the characteristics of patients such as age, race and sex [4]. Variability in the methodology used for data collection and reporting, and different definitions of ATDH may also contribute to the wide range of incidence rates.

The proposed genetic risk factors for ATDH include polymorphisms of the N-acetyltransferase 2 (*NAT2*), cytochrome P450 2E1 (*CYP2E1*), and glutathione s-transferase mu 1 (*GSTM1*) genes, which code for drug-metabolising enzymes. Several studies have investigated the association between these genetic polymorphisms and ATDH [5–24]. These polymorphisms may affect the activation ability of the enzymes, altering the chemical modification of anti-tuberculosis drugs and their metabolites in the liver, leading to hepatic adverse reactions [25]. Toxic metabolites may also cause other toxicity events, such as peripheral neuropathy and maculopapular eruption, although the majority of evidence on the pharmacogenetics of anti-tuberculosis drugs focuses on hepatotoxicity.

Isoniazid is the anti-tuberculosis drug for which the genetic contribution to ATDH is most studied and best understood. Specifically, it is thought that *NAT2* acetylator status may be associated with increased risk of isoniazid-related hepatic adverse reactions, as *NAT2* is one of the main enzymes involved in the metabolism of isoniazid in the liver. There are three phenotypes of acetylator status. Individuals who are slow *NAT2* acetylators acetylate isoniazid slowly, resulting in high plasma drug levels. This may be beneficial for treatment efficacy, but slow acetylators may also experience an accumulation of toxic metabolites as part of the metabolic activation of acetylhydrazine to harmless diacetylhydrazine. Isoniazid suppresses the acetylation of acetylhydrazine, hence producing more toxic metabolites, which contributes to the increased risk of isoniazid hepatitis that slow acetylators experience [26]. Fast acetylators have lower plasma drug levels, and so, treatment may be not only less effective but also less toxic, and intermediate acetylators fall between these two extremes.

Several studies have investigated the association between *NAT2* genetic variants and hepatotoxicity in patients treated with isoniazid or isoniazid-containing regimens [5–18], with most studies reporting significant associations between NAT genetic variants and ATDH. However, there is no set method for defining acetylator status by genotyping, as acetylator status is governed by polymorphisms in a number of alleles on the *NAT2* gene, making the genetic definition of acetylator status difficult to standardise. Cai et al. (2012) [27] defined individuals without a copy of the wild-type NAT2*4 allele as slow acetylators, and all other individuals as rapid acetylators, whereas Wang et al. [28] considered individuals with two copies of rapid *NAT2* acetylator alleles (NAT2*4, NAT2*11A, NAT2*12A, NAT2*12B, NAT2*12C, NAT2*13) to be rapid acetylators, individuals with no copies to be slow acetylators, and those with one copy to be intermediate acetylators.

Following *NAT2* in the disposition of isoniazid is *CYP2E1*. It has been reported that isoniazid inhibits *CYP2E1* activity less in individuals with the *CYP2E1* *1A/*1A genotype than in those with other genotypes [20]. Consequently, individuals with the *CYP2E1* *1A/*1A genotype have higher *CYP2E1* activity, resulting in more hepatotoxins. Some studies have investigated the association between *CYP2E1* genetic variants and hepatotoxicity in patients treated with isoniazid or isoniazid-containing regimens, although results from these studies are often conflicting [13–22].

Finally, GST is an enzyme that plays a crucial role in the detoxification process, and it has been hypothesised that individuals with null *GSTM1* or *GSTT1* genotypes may detoxify the toxic metabolites from earlier in the metabolic pathway of isoniazid less efficiently [27]. There has been some investigation into the association between *GSTM1* and *GSTT1* genotypes and ATDH [18, 22–24], and although significant associations are reported by some studies, the evidence base remains highly uncertain.

Rifampicin and pyrazinamide have also been reported to be hepatotoxic [29, 30]; however, the mechanisms for rifampicin-induced hepatotoxicity and pyrazinamide-induced hepatotoxicity are unknown and unpredictable [4]. Some speculated genetic variants, such as *SLCO1B1*, have been linked to toxicity with rifampicin, although limited data are available to support this claim [29]. No hepatotoxicity has been described for ethambutol.

The purpose of this systematic review and meta-analysis is to evaluate the current evidence on the effect of genetic variants on anti-tuberculosis drug-related toxicity in TB patients receiving anti-tuberculosis drugs. Meta-analyses investigating the pharmacogenetics of anti-tuberculosis agents and toxicity outcomes have been published previously [25, 27, 28, 31–38]. However, conclusions from these meta-analyses are conflicting. For example, Cai et al. (2012) [27] and Du et al. [33] found a significant association between the slow acetylator *NAT2* genotype (i.e., individuals who did not have the NAT2*4 allele) and increased risk of anti-tuberculosis drug-induced liver injury (ATDILI); however, Sun et al. [25] investigated the same genotype and found no significant association. Furthermore, Cai et al. (2012) [27] found a significant association between *CYP2E1* c1/c1 genotype and ATDILI for East Asian populations only, whereas Deng et al. [32], Sheng et al. [35] and Sun et al. [25] identified a significant association regardless of ethnicity. A summary of the findings of the previously conducted meta-analyses is provided in Table 1.

**Table 1:** Summary of findings of previously conducted meta-analyses

Review	Findings			
	*NAT2* gene	*CYP2E1* gene	*GSTM1* gene	*GSTT1* gene
Cai et al. (2012) [27]	Slow *NAT2* genotype (without wild-type NAT2*4 allele) significantly increases the risk of ATDILI	*CYP2E1* c1/c1 genotype significantly increases the risk of ATDILI among East Asian patients. This result is non-significant for the wider population	*GSTM1* null genotype significantly increases risk of ATDILI	*GSTT1* null genotype has no effect on ATDILI risk
Cai et al. (2015) [31]	–	–	The *GSTM1* present genotype is significantly associated with decreased risks of ATDILI and schizophrenia	The *GSTT1* present genotype is significantly associated with a high risk of schizophrenia, but not with ATDILI according to the fixed-effect model
Deng et al. (2012) [32]	–	*CYP2E1* c1/c1 genotype significantly increases the risk of ATDILI. When stratifying for study population, significant results are observed in Chinese and Korean populations	–	–
Du et al. (2013) [33]	Slow acetylator genotype of *NAT2* (without wild-type NAT2*4 allele) significantly increases risk of ATDH. When stratifying for study population, this result is significant for East Asian, South Asian, Brazilian and Middle Eastern populations, but not for Caucasian populations	–	–	–
Li et al. (2013) [34]	–	–	ATDH risk is significantly increased in *GSTM1* null genotypes. Subgroup analyses show that the association is significant in patients receiving HRZE or HRZES, and East Asians. The opposite result is observed for patients treated with HR	The *GSTT1* polymorphism is not associated with ATDH risk, except in patients receiving HRZ
Sheng et al. (2014) [35]	–	*CYP2E1* c1/c1 genotype significantly increases risk of ATDH. *CYP2E1* D/D genotype is not significantly associated with ATDH. *CYP2E1* c1/c1 genotype patients who are *NAT2* slow acetylators have significantly higher risk of ATDH than *CYP2E1* c1/c1 genotype patients who are *NAT2* fast or intermediate acetylators	–	–
Shi et al. (2015)[36]	Slow acetylator status is associated with increased risk of ATDILI compared with fast and intermediate acetylators when standard dose of isoniazid is administered	–	–	–
Sun et al. (2008) [25]	Slow *NAT2* genotype (without wild-type NAT2*4 allele) does not significantly increase risk of ATDILI. A significant effect is demonstrated among Asian populations	*CYP2E1* c1/c1 genotype is significantly associated with an increased risk of ATDILI	*GSTM1* null genotype significantly increases risk of ATDILI	*GSTT1* null genotype has no effect on ATDILI risk
Tang et al. (2013) [37]	–	–	*GSTM1* null genotype is significantly associated with increased risk of ATDILI. Stratified analyses demonstrate significant associations in Chinese populations and when restricting to studies with case numbers >57	No statistically significant association is observed between *GSTT1* null genotype, or *GSTM1*/*GSTT1* interaction and risk of ATDILI
Wang et al. (2012) [28]	Individuals homozygous for rapid *NAT2* acetylator alleles (NAT2*4, NAT2*11A, NAT2*12A, NAT2*12B, NAT2*12C, NAT2*13) are rapid acetylators; individuals homozygous for slow acetylator alleles are slow acetylators, and heterozygous individuals are intermediate acetylators. Slow acetylators have significantly higher risk of ATDILI than other acetylators	–	–	–
Wang et al. 2016 [38]	–	*CYP2E1* c1/c1 genotype is significantly associated with an increased risk of ATDH. *CYP2E1* D/D genotype is not significantly associated with ATDH. When stratifying for study population, significant results are observed in East Asian populations only	–	–

*ATDH* anti-tuberculosis drug-induced hepatotoxicity, *ATDILI* anti-tuberculosis drug-induced liver injury, *H* isoniazid, *R* rifampicin, *Z* pyrazinamide, *E* ethambutol, *S* streptomycin

Despite the informative nature of the previously conducted meta-analyses, there are several methodological limitations to these reviews.

- Cai et al. (2012) [27] only included studies that provided genotype distribution information in both cases and controls, or the odds ratio (OR) with its 95% confidence interval (CI) and *p* value. It is highly likely that some papers would not report either of these details, and therefore, key evidence may have been omitted from this systematic review. Li et al. [34] and Shi et al. [36] both also excluded papers without genotype data for both cases and controls. Deng et al. [32], Du et al. [33], Sheng et al. [35], Tang et al. [37], Wang et al. [28] and Wang et al. [38] also excluded papers if reported data were insufficient to calculate ORs.
- Cai et al. (2012) [27] excluded three studies that were “non-RCTs”. No definition of “non-RCT” was provided, although we assume the authors are referring to studies that are not randomised controlled trials (RCTs). Pharmacogenetic studies are likely to be non-randomised studies, such as case–control or cohort designs, so this is not a valid reason for excluding studies, and important evidence may have been omitted from the meta-analysis.
- Cai et al. (2015) [31], Deng et al. [32], Li et al. [34], Sheng et al. [35], Shi et al. [36], Sun et al. [25], Tang et al. [37] and Wang et al. [28] all included only case–control studies. While it does not seem as though any studies were excluded on this basis for most of the reviews, Sun et al. [25] did not provide a literature search map, and so, it is unclear whether any studies were excluded on this basis. Furthermore, it is not clear from the literature search map provided in Cai et al. (2015) [31] whether any non-case–control studies were excluded. Genetic association data may come from non-case–control studies, such as RCTs.
- Cai et al. (2012) [27], Cai et al. (2015) [31], Du et al. [33] and Li et al. [34] did not assess study quality, which is a key component of any systematic review.
- Sun et al. [25] did assess study quality using Little’s checklist [39]; however, the review authors deemed two studies to be of poor quality but did not investigate the impact of including these poor quality studies in the meta-analyses.

In order to overcome these limitations, our systematic review and meta-analysis will not exclude papers if sufficient data are not reported; instead, we will contact study authors to obtain the required information. Our review will include both non-RCTs and RCTs if relevant data are measured. We will also assess studies with respect to their quality and strength of evidence and investigate the impact of including poor-quality studies by conducting sensitivity analyses.

In addition, the scope of our review will be wider than the previously conducted meta-analyses. Cai et al. (2012) [27] and Sun et al. [25] excluded studies if they did not report hepatotoxicity. Our meta-analysis will consider all effects of genetic polymorphisms on all toxicity outcomes. Furthermore, all previously conducted meta-analyses restricted inclusion to those studies that considered specific genes; our review will include all pharmacogenetic studies in this field. In addition, as the most recent of these meta-analyses included papers up to November 2013, our review may include additional evidence from studies published in the past 3 years, which would not have been included in any of the previously published meta-analyses.

Our review and meta-analysis will update and add to the existing research which has previously been conducted in this field.

## Methods

This protocol has been prepared in accordance with the Preferred Reporting Items for Systematic Review and Meta-analysis Protocols (PRISMA-P) statement [40], as provided in Additional file 1.

### Selection criteria

Types of studies: We expect that most studies identified will be retrospective or prospective cohort studies; however, we will also include case–control studies and RCTs. Case–control studies may have been conducted to compare patients who experienced toxicity events with those who did not. RCTs may have been conducted to investigate the effectiveness of genotyping TB patients before prescribing anti-tuberculosis agents. In this case, we would include data from patients in the genotyped arm of the trial. We may also identify RCTs in which none of the patients were originally genotyped, but a subsequent post hoc genetic analysis was conducted in a subset of patients or in the whole trial population, and we will also include these studies.

Types of participants: We will include studies that recruited TB patients who were either already established on anti-tuberculosis treatment or commencing treatment and who were genotyped to investigate the effect of genetic variants on anti-tuberculosis drug-related toxicity.

Types of outcomes: We will include studies that measured any toxicity outcomes.

### Inclusion/exclusion criteria

Studies will have to satisfy the following inclusion criteria to be included in our review.

- The majority of patients should have been TB patients receiving anti-tuberculosis treatment (at least one of isoniazid, rifampicin, pyrazinamide or ethambutol).
- Patients should have been genotyped.
- Association between at least one genetic variant and at least one toxicity outcome should have been assessed.

Studies will be excluded if either of the following exclusion criteria is met.

- The only outcomes were pharmacokinetic outcomes.
- A case study design was used.

### Outcomes

The primary outcome of our review will be hepatotoxicity by any definition. Secondary outcome will be all other toxicity outcomes.

### Identification of studies

A search specialist will design the search strategy and will search for relevant studies in MEDLINE, PUBMED, EMBASE, BIOSIS and Web of Science. The following Medical Subject Headings (MeSH) words will be used as part of our search strategy: antitubercular agents, antitubercular drugs, isoniazid, rifampicin, pyrazinamide, ethambutol, genetic polymorphism, genetic susceptibility, pharmacogenetics, pharmacogenomics, genetic association study, genetic association analysis, tuberculosis. Details of the planned search strategy are provided in Additional file 2.

We will hand search reference lists from relevant studies and contact experts in the field, in order to identify further eligible studies. We will include studies published in English only. We will not restrict by year of publication or publication status.

Once we have a complete list of studies identified by the search, literature search results will be imported to Covidence [41], an Internet-based software programme that facilitates collaboration among reviewers during the study selection process. We will remove duplicates, and one author (MR) will independently scan the abstracts of these studies to remove any obviously irrelevant studies. A second author (AJ or JK) will independently screen a sample of 10% of studies.

We will obtain the full text for each potentially relevant study. One reviewer (MR) will independently assess eligibility for inclusion in the review based on the inclusion and exclusion criteria. A second author (AJ or JK) will independently assess a sample of 10% of studies for inclusion. We will seek additional information from study authors where necessary to resolve questions about eligibility. Any disagreements between the two reviewers will be resolved through discussion and by consulting a third author if necessary.

For conference abstracts that satisfy our inclusion criteria, and for conference abstracts for which eligibility is unclear, we will attempt to locate the full journal article by searching for the studies online or by contacting the conference abstract authors, in order to guide our eligibility judgement. If we are unable to locate the full paper, or if the authors confirm that the work has not been published in full, we will exclude these studies. If we identify the full paper for an eligible study, we will add this study to our included studies.

### Data collection

Two authors will independently extract data in accordance with the methods outlined in the Cochrane Handbook for Systematic Reviews of Interventions [42] and as recommended in The HuGENet HuGE Review Handbook [43]. We will design a data extraction form and pilot this using a random sample of the studies to be reviewed. We will collect data on study design, participant characteristics, treatment regimen and outcomes. The form will also enable us to assess study quality, as described below. We will contact authors of the studies if we require additional information in order to assess study quality or the appropriateness of pooling data from different studies, and if outcome data required to calculate effect sizes are missing. Any disagreements between the two reviewers will be resolved through discussion and by consulting a third author if necessary.

### Assessment of study quality

Two authors will use the criteria in Table 2, as developed in a previous meta-analysis of pharmacogenetics studies [44], to assess the quality of each included study, at study level, in relation to specific genetic association study issues. Any disagreements between the two reviewers will be resolved through discussion and by consulting a third author if necessary.

**Table 2:** Criteria for quality assessment of genetic association studies

Issue	Assessment criteria
1. Choosing the genes/single-nucleotide polymorphisms (SNPs) to genotype	Was a literature review undertaken and the findings summarised? Are reasons given for choosing the genes and SNPs genotyped? If reasons include previous association studies, are key details from these provided? If reasons include functional studies are supporting data provided? Is method to adjust for multiple testing described? Are precise *p* values provided for all associations?
2. Sample size	What is the sample size? Are details given of how the sample size was calculated? Are details given of the *a priori* power to detect effect sizes of varying degrees?
3. Study design	What is the study design? If study is case–control, are the two groups clearly defined? If study is case-control, were they genotyped in mixed batches?
4. Reliability of genotypes	Is the genotyping procedure described? Are the primers described? Were quality control methods used and described? Were findings from quality control methods, if used, described? Are any genotype frequencies previously reported quoted? Were genotyping personnel blinded to outcome status? If human inference required, was this independently undertaken by at least two people?
5. Missing genotype data	Is extent of missing data summarised? Where extent is summarised are reasons for missing data given? Are checks for missingness at random reported? Are missing genotype data imputed? Does paper quote number of patients contributing to each analysis? If paper does quote number of patients contributing to analyses, does this agree to sample size?
6. Population stratification	Are tests undertaken for cryptic population stratification? If so, are results quoted? Is cryptic population stratification adjusted for in the analyses?
7. Hardy–Weinberg equilibrium (HWE)	What test is undertaken to check for HWE? Where test undertaken, is *p* value threshold applied to determine deviation from HWE quoted? Where test undertaken, are SNPs deviating from HWE highlighted? Where test undertaken, and some SNPs found to deviate, are steps taken to explore deviation from HWE reported? Where test undertaken, and some SNPs found to deviate, are deviating SNPs excluded from further analysis?
8. Mode of inheritance	Is a specific mode of inheritance assumed? If so which? Is justification provided for assumptions made regarding mode of inheritance (if no mode or a specific mode is assumed)? If no mode of inheritance is assumed does the paper explain limitations of this? If several analyses undertaken under different assumptions, are they adjusted for multiple testing?
9. Choice and definition of outcomes	Does the paper clearly define all outcomes investigated? Is justification provided for the choice of outcomes? Are results shown for all outcomes mentioned?
10. Adherence to treatment	Is adherence to treatment measured? If adherence is measured, are adjustments for non-adherence made in the analyses?

*HWE* Hardy–Weinberg equilibrium, *SNP* single-nucleotide polymorphism

### Data synthesis

Where possible, we will pool the estimates of effect for each genotype on each outcome. We will consider the differences in definition of hepatotoxicity or other toxicity outcomes and perform stratified analyses if it is not clinically meaningful to combine results for different definitions of outcomes in meta-analysis.

We will decide on which genotype groups to compare based on the primary papers. If primary papers vary in terms of which genotype groups they compare, we will use methods proposed in the literature to combine these [45].

As we do not know the genetic model underlying the effects of the various genetic polymorphisms, we will implement a “genetic model-free” [46] approach when pooling effect estimates. This approach assumes a common genetic model across all studies included in a meta-analysis, which is estimated from the data. If the data suggest that this assumption is violated, we will use a bivariate meta-analysis model to conduct joint pairwise comparisons. This model accounts for the fact that the effect measures for homozygous-mutant versus homozygous wild-type genotypes and for heterozygous versus homozygous wild-type genotypes will be correlated.

We will perform meta-analyses using R, generating ORs and 95% CIs for dichotomous data, and mean differences and standard errors for continuous data. Forest plots will be produced. We will assess heterogeneity by visually examining the forest plots and by considering the *I* ^*2*^ statistic [47]. If heterogeneity is minimal, we will perform meta-analysis using fixed-effects models. If considerable heterogeneity is observed, we will attempt to explain this heterogeneity using subgroup analyses. However, if some unexplained heterogeneity remains, we will use random-effects models for meta-analysis. Finally, if heterogeneity is substantial and cannot be explained by subgroup analyses, we may decide that it is not appropriate to perform any meta-analysis of the data. If included studies are too heterogeneous to combine in a meta-analysis, we will display the results of included studies in a forest plot but suppress the pooled estimate, and we will summarise the results of the included studies descriptively in the text and tables of the review.

### Investigation of heterogeneity and subgroup analysis

We will perform stratified analyses by ethnic groups, and if effect estimates are found to be similar across different ethnic groups, then we will pool these results. We may use meta-regression to investigate the effect of continuous variables on treatment effect or, if it is appropriate, to investigate the effects of multiple factors in the same analysis. We will not carry out meta-regression if fewer than 10 studies are included in the meta-analysis.

If heterogeneity exists, we will investigate this heterogeneity by performing subgroup analyses according to ethnic groups, study design, outcome definitions, treatment regimens, and date of study publication, as early studies often overestimate effects.

### Selective reporting

We will attempt to identify cases of selective reporting within the included studies by constructing an outcome matrix [48], with the review outcomes and outcomes reported by included studies listed. We will scrutinise studies with missing outcome data and will contact the authors of the study if a study does not report results for the outcomes of interest. We will not exclude studies on the basis of missing outcome data [49]. We will perform sensitivity analyses to assess the robustness of the conclusions of meta-analyses to outcome reporting bias [50–52]. We will also scrutinise studies in order to identify possible selective reporting with regard to reporting results for all pre-specified genetic variants.

### Publication bias

We will produce funnel plots if enough studies are available (>10 studies), in order to assess the risk of publication bias.

### Sensitivity analyses

We will perform sensitivity analyses excluding poor-quality studies, in order to assess the impact of these studies on pooled effect estimates.

### Confidence in cumulative evidence

The strength of the body of evidence will be assessed according to the Venice interim criteria [53].

## Discussion

We do not anticipate that there will be any practical or operational issues in the implementation of this protocol. There is a key issue that we will consider when performing our systematic review and meta-analysis, and interpreting the results––namely, the scope of the available data with regard to geographical location. There is a great deal of genetic variability across the African continent, where TB is endemic, and there is very little mapping of pharmacogenomic polymorphisms in African populations. Although we will assess the quality of included studies with regard to possible cryptic population stratification, and we will perform subgroup analyses by ethnic group to investigate how estimates of effect for genetic variants on toxicity outcomes vary according to population, it may be the case that results are still not representative of the global population most affected by TB.

## Additional files

Additional file 1: PRISMA-P 2015 checklist. Containing the populated PRISMA-P checklist table, demonstrating how this protocol has been prepared in accordance with the PRISMA-P statement. (DOCX 33 kb)

Additional file 2: Search strategy. Outlining the search terms and search strategy that we will use to identify studies to include in our systematic review and meta-analysis. (DOCX 20 kb)

## Abbreviations

ATDH: Anti-tuberculosis drug-induced hepatotoxicity
ATDILI: Anti-tuberculosis drug-induced liver injury
CI: Confidence interval
*CYP2E1*: Cytochrome P450 2E1
*GSTM1*: Glutathione s-transferase mu 1
HWE: Hardy–Weinberg equilibrium
MeSH: Medical Subject Headings
*NAT2*: N-acetyltransferase 2
OR: Odds ratio
RCT: Randomised controlled trial
SNP: Single-nucleotide polymorphism
TB: Tuberculosis
WHO: World Health Organization
