# Supplementary material for: Influence of genetic variants on toxicity to anti-tubercular agents: a systematic review and meta-analysis (protocol)
Source: Syst Rev. 2017 Jul 13;6:142. doi: 10.1186/s13643-017-0533-4 (PMC5508765; doi:10.1186/s13643-017-0533-4)
Supplement: Supplementary file 2 — Search strategy. Outlining the search terms and search strategy that we will use to identify studies to include in our systematic review and meta-analysis. (DOCX 20 kb) [file 13643_2017_533_MOESM2_ESM.docx]

**Additional File 2: Search Strategy**

***Databases to be searched:***

- MEDLINE (Ovid) and MEDLINE In-Process (Ovid)
- EMBASE (Ovid)
- PubMed
- Web of science
- Biosis

***Search strategies:***

Database: Web of Science and Biosis

| # 1 | **TITLE:** ((((Antitubercul* or tuberculos* or TB) Near/4 (agent* or drug* or antibiotic* or medicine* or medication* or treatment*)))) |
| --- | --- |
| # 2 | **TITLE:** (((aminosalicylic acid or diarylquinoline* or ethambutol* or ethionamide* or isoniazid* or prothionamide* or pyrazinamide* or thioacetazone* or capreomycin* or cycloserine* or enviomycin* or rifabutin* or rifampin* or viomycin*))) |
| # 3 | **TITLE:** ((((genetic* or gene*) near/3 (suscept* or predisposit* or anticipat*)))) |
| # 4 | **TITLE:** (((single* near/2 nucleotid* near/2 polymorph*))) |
| # 5 | **TITLE:** ((((SNP or Genotyp* or Phenotyp* or Allele* or Pharmacogenet* or Pharmacogenom* or Polymorph*)))) |
| # 6 | **TITLE:** ((((gene* or genetic*) near/5 (mutat* or variant*)))) |
| # 7 | **TITLE:** ((((TB or Tuberculosis* or Antitubercul*)))) |
| # 8 | #2 OR #1 |
| # 9 | **TITLE:** ((((Genetic or gene*) near/2 associat* near/2 (studies or study or analys*)))) |
| #10 | #9 OR #6 OR #5 OR #4 OR #3 |
| **# 11** | **#10 AND #8 AND #7** |

Database: Medline

| 1 | antitubercular agents/ or aminosalicylic acid/ or diarylquinolines/ or ethambutol/ or ethionamide/ or isoniazid/ or prothionamide/ or pyrazinamide/ or thioacetazone/ or antibiotics, antitubercular/ or capreomycin/ or cycloserine/ or enviomycin/ or rifabutin/ or rifampin/ or viomycin/ |
| --- | --- |
| 2 | ((Antitubercul* or tuberculos* or TB) adj4 (agent* or drug* or antibiotic* or medicine* or medication* or treatment*)).tw. |
| 3 | (aminosalicylic acid or diarylquinoline* or ethambutol* or ethionamide* or isoniazid* or prothionamide* or pyrazinamide* or thioacetazone* or capreomycin* or cycloserine* or enviomycin* or rifabutin* or rifampin* or viomycin*).tw. |
| 4 | 1 or 2 or 3 |
| 5 | Polymorphism, Genetic/ |
| 6 | genetic predisposition to disease/ or anticipation, genetic/ |
| 7 | Pharmacogenetics/ |
| 8 | Genetic Association Studies/ |
| 9 | ((Genetic or gene*) adj2 associat* adj2 (studies or study or analys*)).tw. |
| 10 | ((genetic* or gene*) adj3 (suscept* or predisposit* or anticipat*)).tw. |
| 11 | Polymorphism, Single Nucleotide/ |
| 12 | (single* adj2 nucleotid* adj2 polymorph*).tw. |
| 13 | (SNP or Genotyp* or Phenotyp* or Allele* or Pharmacogenet* or Pharmacogenom* or Polymorph*).tw. |
| 14 | ((gene* or genetic*) adj5 (mutat* or variant*)).tw. |
| 15 | Genotype/ or Phenotype/ or Alleles/ |
| 16 | or/5-15 |
| 17 | exp Tuberculosis/ |
| 18 | (TB or Tuberculosis*).tw. |
| 19 | Antitubercul*.tw. |
| 20 | or/17-19 |
| 21 | 4 and 16 and 20 |
| 22 | animal/ not human/ |
| **23** | **21 not 22** |

Database: Embase

| 1 | antitubercular agents/ or aminosalicylic acid/ or diarylquinolines/ or ethambutol/ or ethionamide/ or isoniazid/ or prothionamide/ or pyrazinamide/ or thioacetazone/ or antibiotics, antitubercular/ or capreomycin/ or cycloserine/ or enviomycin/ or rifabutin/ or rifampin/ or viomycin/ |
| --- | --- |
| 2 | ((Antitubercul* or tuberculos* or TB) adj4 (agent* or drug* or antibiotic* or medicine* or medication* or treatment*)).tw. |
| 3 | (aminosalicylic acid or diarylquinoline* or ethambutol* or ethionamide* or isoniazid* or prothionamide* or pyrazinamide* or thioacetazone* or capreomycin* or cycloserine* or enviomycin* or rifabutin* or rifampin* or viomycin*).tw. |
| 4 | 1 or 2 or 3 |
| 5 | Polymorphism, Genetic/ |
| 6 | genetic predisposition to disease/ or anticipation, genetic/ |
| 7 | Pharmacogenetics/ |
| 8 | Genetic Association Studies/ |
| 9 | ((Genetic or gene*) adj2 associat* adj2 (studies or study or analys*)).tw. |
| 10 | ((genetic* or gene*) adj3 (suscept* or predisposit* or anticipat*)).tw. |
| 11 | Polymorphism, Single Nucleotide/ |
| 12 | (single* adj2 nucleotid* adj2 polymorph*).tw. |
| 13 | (SNP or Genotyp* or Phenotyp* or Allele* or Pharmacogenet* or Pharmacogenom* or Polymorph*).tw. |
| 14 | ((gene* or genetic*) adj5 (mutat* or variant*)).tw. |
| 15 | Genotype/ or Phenotype/ or Alleles/ |
| 16 | or/5-15 |
| 17 | exp Tuberculosis/ |
| 18 | (TB or Tuberculosis*).tw. |
| 19 | Antitubercul*.tw. |
| 20 | or/17-19 |
| 21 | 4 and 16 and 20 |
| 22 | animal/ not human/ |
| **23** | **21 not 22** |

Database: PubMed (will be searched for studies published within 6 months prior to the search date)

| #1 | Search (((Antitubercul* or tuberculos* or TB))) AND ((agent* or drug* or antibiotic* or medicine* or medication* or treatment*)) |
| --- | --- |
| #2 | Search ((aminosalicylic acid or diarylquinoline* or ethambutol* or ethionamide* or isoniazid* or prothionamide* or pyrazinamide* or thioacetazone* or capreomycin* or cycloserine* or enviomycin* or rifabutin* or rifampin* or viomycin*)) |
| #3 | Search (#1 or #2) |
| #4 | Search ((((Genetic or gene*) near/2 near/2 ))) AND associat*) AND ((studies or study or analys*)) |
| #5 | Search (((genetic* or gene*))) AND ((suscept* or predisposit* or anticipat*)) |
| #6 | Search ((single*) AND nucleotid*) AND polymorph* |
| #7 | Search ((SNP or Genotyp* or Phenotyp* or Allele* or Pharmacogenet* or Pharmacogenom* or Polymorph*)) |
| #8 | Search (((gene* or genetic*))) AND ((mutat* or variant*)) |
| #9 | Search (#4 or #5 or #6 or #7 or #8) |
| #10 | Search (((((TB or Tuberculosis* or Antitubercul*))))) |
| **#11** | **Search (#3 and #9 and #10)** |
